# Effectiveness of Non-Pharmacological Interventions for Dementia among the Elderly: A Randomized Controlled Trial

**DOI:** 10.3390/geriatrics9020052

**Published:** 2024-04-18

**Authors:** Huong Thu Vu, Hung Trong Nguyen, Anh Trung Nguyen

**Affiliations:** 1 Department of Geriatrics, Hanoi Medical University, Hanoi 100000, Vietnam; vuthuhuong93@gmail.com; 2 National Geriatric Hospital, Hanoi 100000, Vietnam; nguyentronghung67@gmail.com; 3 Neurology Department, Hanoi Medical University, Hanoi 100000, Vietnam

**Keywords:** non-pharmacological multifactorial intervention, dementia, mental health

## Abstract

(1) Background: Up until now, there is still no medicine that can cure dementia, but there are some that can only help slow down the progression of the disease and reduce some symptoms. Pharmacological interventions for dementia have many side effects and are expensive, so non-pharmacological treatments for dementia become more urgent. This study aimed to evaluate the effectiveness of multifactorial non-pharmacological interventions in dementia patients; (2) Methods: This is a randomized controlled trial conducted in Hai Duong from July 2021 to December 2022. Selected subjects included 88 patients diagnosed with very mild, mild, and moderate dementia, of whom 44 patients were assigned to the intervention group and 44 patients to the control group; (3) Results: For the effectiveness of the non-pharmacological multifactorial intervention on depression severity: in the intervention group, the GDS 15 depression score decreased from 4.8 to 2.9, while, in the control group, the GDS 15 depression score increased by 1.3 points after six months of no intervention. For the effect of the non-pharmacological multifactorial intervention on the level of sleep disturbance, in the intervention group, the PSQI sleep disturbance score decreased by nearly half (from 10.2 to 5.6), while, in the control group, this trend was not clear. For the effect of the non-pharmacological multifactorial intervention on daily functioning: in the intervention group, the ADL and IADL scores improved (1.02 ± 1.32 and 1.23 ± 1.75), while, in the control group, the ADL and IADL scores decreased (0.93 ± 1.2 and 0.98 ± 2.19). For the effect of the non-pharmacological multifactorial intervention on quality of life: in the intervention group, the EQ-5D-5L scores improved (0.17 ± 0.19), while, in the control group, the EQ-5D-5L scores decreased (0.20 ± 0.30); (4) Conclusions: Non-pharmacological multifactorial interventions, including physical activity, cognitive training, listening to educational lectures, and organizing miniature social models, have been shown to improve mental health, self-control, and quality of life.

## 1. Introduction

Dementia is a chronic, progressive syndrome that impairs cognitive domains and affects the ability to exercise autonomy in daily activities [1]. The disease is currently the seventh leading cause of death, and the main cause of disability and loss of autonomy in the elderly [1]. According to estimates, the number of people with dementia in the world will increase from 57.4 million in 2019 to 152.8 million in 2050 [2]. This not only affects people with dementia but also caregivers, families, communities, and society. and increases the financial burden on the health system. Medical care costs for dementia amounted to about 800 billion USD (accounting for 1% of global GDP) in 2015 and are expected to reach 2000 billion USD by 2030 [3].

Vietnam is not an exception to global health trends. The United Nations Economic and Social Commission for Asia and the Pacific (2017) reported that Vietnam’s population aging trend is forecasted to be the fastest in the region [4]. According to the 2019 Population and Housing Census, the population of elderly people in Vietnam in 2019 was 11.41 million (accounting for 11.86% of the total population). This number is forecasted to reach 31.69 million people (accounting for 27.11% of the total population) by 2069 [5]. 

Until now, there is no medicine that can cure dementia, but there are some that can only help slow the progression of the disease and reduce some symptoms. Moreover, drug interventions have many side effects and are expensive, so research on non-drug treatments in dementia has become more urgent [6]. Recently, many dementia prevention classes have been established by local governments across Japan, showing that these programs are initially effective [7]. The results of this program were published in Japan by Minoru Kouzuki and colleagues. The program has the advantage of being inexpensive, easily administered, and standardized, and does not require highly trained staff, thereby potentially enabling its widespread application at the community level [8]. 

Several studies have reported that monotherapy physical or cognitive exercise programs may help slow cognitive decline in patients with dementia; however, the benefits are often limited and short-lived [9,10]. Therefore, the current trend in dementia intervention is multifactorial, including diet control, exercise, active cognitive training, and vascular risk monitoring, aiming to maximize improvements in cognitive and neurocognitive functions [11]. In a low- and middle-income country, besides the health system in Vietnam that is not yet fully developed in the field of geriatrics and mental health, the ability to diagnose and intervene without drugs for dementia is still limited [12]. Hence, this study aims to evaluate the effectiveness of non-pharmacological multifactorial interventions in Vietnamese dementia patients.

## 2. Materials and Methods

### 2.1. Participants

We included patients with dementia diagnosed according to DSM-5 criteria, aged 60 years or older, and categorized as having very mild, mild, or moderate dementia based on the CDR [13]. We excluded patients who desired to treat dementia with medication to achieve treatment goals; had joint problems preventing participation in physical activities such as knee osteoarthritis and severe lumbar spine degeneration; answered ‘Yes’ to one or more questions about their cardiovascular history during the interview; and those who refused to participate in the study. 

### 2.2. Study Design and Setting

A randomized controlled trial was conducted in Hai Duong province (Vietnam) from July 2021 to December 2022. Hai Duong province is located in the center of the Red River Delta, is one of the provinces with the largest industry attracting foreign investment in Vietnam, and has favorable conditions in transportation, agriculture, and industry [14]. Hai Duong has a combination of rural and semi-urban areas, similar to the general level in Vietnam, thereby improving the generalizability of the findings: more than 2/3 of Vietnam’s population live in rural areas. This allowed us to examine rural and semi-urban conditions as potential moderators of intervention effects at the commune level [15]. A list of elderly people was made in Thanh Mien and Gia Loc districts of Hai Duong province. Selection criteria for the sample was applied to commune health centers of Thanh Mien and Gia Loc districts of Hai Duong province.

The sample size for the intervention study was estimated based on the formula recommended by the World Health Organization for the difference of two proportions:

$$n=\frac{{\left\{Z_{1-\alpha /2}\sqrt{2\bar{P}(1-\bar{P}})+Z_{1-\beta }\sqrt{P_{1}\left(1-P_{1}\right)+P_{2}\left(1-P_{2}\right)}\right\}}^{2}}{\left(P_{1}-P_{2}\right)}$$

where:

n: the minimum sample size of each intervention group and control group;

P_1_: the rate of very mild, mild, and moderate dementia changed in the intervention group, estimated at 5%;

P_2_: the rate of dementia at very mild, mild, and moderate levels changed in the control group, estimated at 12%;

P: average change in dementia rate, P = (P_1_ + P_2_)/2;

$Z_{1-\alpha /2}$: reliability coefficient, corresponding to 95% confidence level $Z_{1-\alpha /2}$ = 1.96;

$Z_{1-\beta }$: desired sample force, corresponding to 80% level, $Z_{1-\beta }$ = 0.93.

We estimated an additional 10% for refusals and loss of follow-up. Prior to the intervention, we assigned the number of research subjects to each group: 25 patients in the intervention group and 25 patients in the control group. Finally, the study recruited 44 patients for the intervention group and 44 patients for the control group.

### 2.3. Survey Instruments

The socioeconomic characteristics we measured included age, gender, education level, marital status, and employment. The diagnosis of dementia among the selected individuals was obtained according to the Diagnostic and Statistical Manual of Mental Disorders, fifth edition (DSM-5) [13,16,17]. Validated Vietnamese questionnaires were used to evaluate the lifestyle characteristics and medical histories. The level of dementia was evaluated using the Clinical Dementia Rating (CDR) Questionnaire in Vietnamese [13]. Physical function test included IPAQ—SF physical activity level scale, OLS 30 s standing test, TUG stand-up-and-go timed test, 6 min 6MW walking test, and functional test with FRt. The level of sleep quality was evaluated according to the Pittsburgh Sleep Quality Index PITTSBURGH (PSQI) [18,19]. Alcohol abuse was evaluated according to the Alcohol Use Disorders Identification Test-Concise (AUDIT-C) [20,21]. Level of physical activity was assessed by the Short Form International Physical Activity Questionnaire (IPAQ-SF) [22,23]. Level of nicotine dependence was assessed according to the Fagerström test for nicotine dependence (FTND) scale [21,24,25,26]. Quality of life was assessed according to the European Quality of Life (EQ-5D-5L) [27]. The Instrumental Activities of Daily Living (IADL) questionnaire [28,29], self-control in daily activities according to the Activities of daily living (ADL) [30,31], and the 15-item Geriatric Depression Scale [32,33] were also used. 

### 2.4. Intervention

Using Block 08 method: Make 08 ballots (04 ballots recording EL—intervention group, 04 ballots recording NC—control group), and put them in a box. Clinic staff were trained in randomization methods and worked completely independently from the researchers. Each patient draws 01 lottery in the box, on which is written the name of the group in which the patient will participate in the study and the nurse will inform the researcher about this.

We applied the intervention content based on previous valid evidence by the author Minoru Kouzuk et al. [8]. The program lasts 24 sessions. The content of a workout session includes (1) 50 min of physical exercise, (2) 20 min of breaks or 20 min of education about dementia and living habits, and (3) 50 min of exercise and cognitive training. The lecture was developed by doctors in the Department of Neurology and Rehabilitation of the National Geriatric Hospital Vietnam and recorded by 3 rehabilitation technicians with extensive experience in care and exercise for patients with dementia. The intervention management team at the commune health station includes 6 commune health workers and 3 students from Hai Duong Medical Technical University. To ensure research ethics, the control group listened to lectures about dementia and living habits that were recorded on DVD and played for 20 min once a week continuously for 6 months. At the same time, the control group also received health checks like the intervention group.

**Figure 1:**
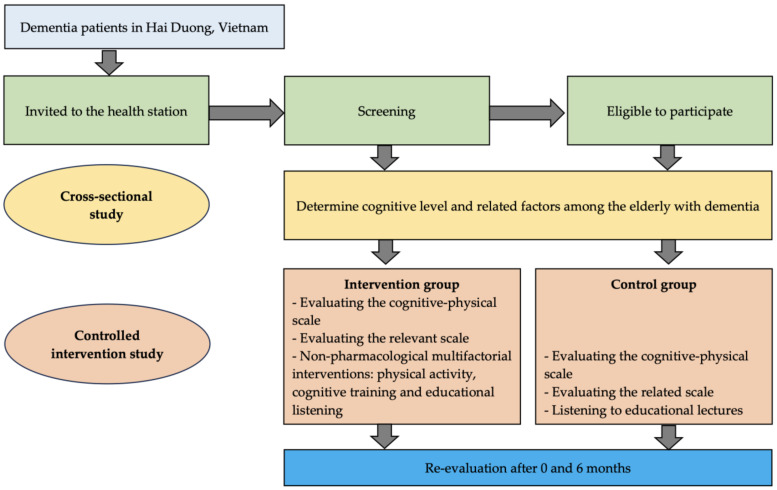
Research diagram.

This study conducted a suggested model that included physical activity, cognitive training, and participation in educational lectures for dementia patients (Figure 1).

### 2.5. Data Analysis

Data were imported and managed by Kobotoolbox software (v2021.2.4). Then, the data were cleaned, processed, and analyzed using STATA 17.0 software. Continuous variables were expressed as mean, and standard deviation (SD) with interquartile ranges (IQRs). Categorical variables were presented as counts with percentage. The McNemar’s test and paired *t*-test was used. We also applied the Poisson regression model with adjusted variance to estimate the prevalence ratio (prevalence ratios—PRs) with 95% confidence interval (95% CI). A *p*-value < 0.05 was considered to be statistically significant.

## 3. Results

As was shown in Table 1, the majority were female in both groups (79.5% in the intervention group and 84.1% in the control group). There were no significant differences in age, occupation before retirement, and marital status between the two groups. The levels of moderate and high physical activity were almost similar (100% in the intervention group and 96.7% in the control group). The degree of dementia was 1.1 ± 0.3 in the intervention group and 1.07 ± 0.3 in the control group. The intervention group had a shorter 30 s stand time than the intervention group (10.3 vs. 12 s), and walked 6 min less (109 vs. 132 m), but had a shorter stand-up-and-walk time (15.5 vs. 14.2 s). However, these differences were not statistically significant (*p* > 0.05).

**Figure 2:**
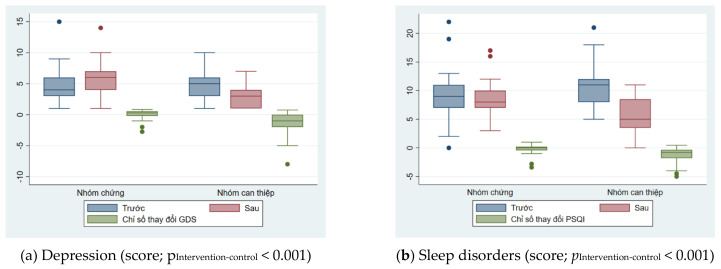
Changes in intervention indicators of depression and sleep disorders between the two groups.

Changes in intervention indicators of depression and sleep disorders between the two groups were illustrated in Figure 2. Depression also showed a similar trend after the intervention. For the intervention group, depression scores decreased by nearly half after intervention, from 4.8 to 2.9. In contrast, for the control group, depression scores increased by 1.3 points after six months of no intervention. The trend of changing the level of sleep disturbance also shows the effectiveness of the intervention. For the intervention group, sleep disturbance scores decreased by nearly half, from 10.2 to 5.6 points. For the control group, this trend is not clear, when the disorder score decreased slightly from 9.0 points to 8.3 points (Table 2).

**Figure 3:**
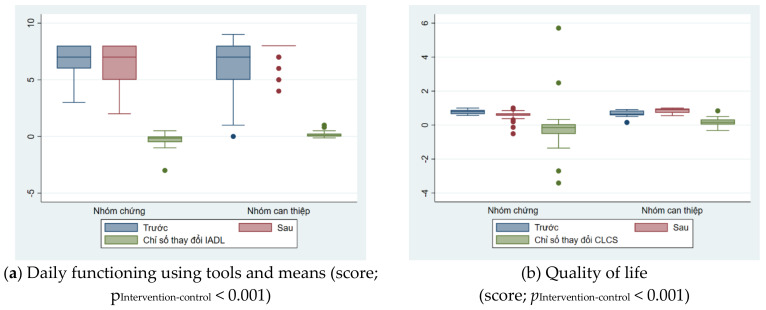
Changes in intervention indicators of daily functioning and quality of life between the two groups.

Table 3 showed the factors of daily functioning and quality of life also had similar trends, showing the effectiveness of the intervention. For the intervention group, scores assessing daily functioning without or with instruments improved after intervention, while these scores decreased in the control group. Changes in intervention indicators of daily functioning and quality of life between the two groups were illustrated in Figure 3.

## 4. Discussion

The intervention program included physical exercise, cognitive training, access to knowledge about dementia, and group-based activities. For the basis of intervention, physical activity helps increase the neuroprotective factor BDNF, reduce inflammatory factors, prevent vascular risk factors, reduce the risk of falls, and improve health. Previous comprehensive studies showed the cognitive effectiveness of physical intervention. However, these results were not consistent; there were many fluctuations in results, and even no cognitive improvement. Therefore, the current trend is to combine physical activity and cognitive training interventions, which helps increase cognitive stimulation. In particular, a recognized risk factor and consequence of dementia is social isolation, which is of increasing concern. To make the intervention optimal, we conduct it in a miniature social model of group practice. The research aimed to validate this claim on Vietnamese subjects by studying the effectiveness of multifactorial non-pharmacological interventions on dementia patients. From the results of the study involving 399 research patients, 88 patients completed 24 follow-up sessions (44 patients in the control group and 44 patients in the intervention group), yielding positive outcomes that could potentially lead to cost savings. This finding supports the recommendation of utilizing non-pharmacological multifactorial interventions for patients diagnosed with dementia. A standardized program, which is cost-effective and does not demand highly specialized monitoring personnel, can be widely implemented in the community.

At the beginning of the intervention, there were no statistically significant differences between the intervention group and the control group in terms of characteristics: age, gender, occupation before retirement, health status. marriage, level of dementia, and level and ability of physical activity. Depression in people with dementia is associated with adverse health outcomes, including a lower quality of life, impaired self-control, and an increased risk of mortality [34,35]. Dementia was also associated with increased suffering, burden, and depression for caregivers [34,36]. In our study, for the intervention group, depression scores decreased by nearly half after intervention, from 4.8 to 2.9. In contrast, for the control group, depression scores increased by 1.3 points after six months of no intervention. Compared with the results of a review that screened 22,138 citations, 256 studies were obtained, equivalent to 28,483 people with dementia. The review found that seven interventions were associated with reduced depressive symptoms compared with usual care, including: cognitive stimulation (mean difference −2.93, 95 CI %: (−4.35)–(−1.52)), cognitive stimulation associated with anticholinesterase use (−11.39, 95% CI: (−18.38)–(−3, 93)), massage and touch therapy (−9.03, 95% CI: (−12.28)–(−5.88)), multifactorial care (−1.98, 95% CI: (−3.80)–(−0.16)), occupational therapy (−2.59, 95% CI: (−4.70)–(−0.40)), and reminiscence therapy (−2.30, 95% CI: (−3.68)–(−0.93)), and the most effective measure was physical activity combined with cognitive stimulation and social interaction (−12.37, 95% CI: 19.01–(−5.36)). Among them, three measures are more effective than some drug interventions—massage and touch therapy, cognitive stimulation combined with anticholinesterase use, and physical activity combined with cognitive stimulation—and have social interactions [37].

Learning about the pathogenesis helps non-pharmacological interventions such as physical activity contribute to improving depressive symptoms in dementia patients. Physical activity helps increase the production of the neurotransmitters dopamine, serotonin [38], and brain-derived neurotrophic factor BDNF [39], contributing to the treatment of depression. Moreover, one of the protective effects of regular exercise is to reduce serum cortisol levels [40]. According to Ouanes, high levels of cortisol in Alzheimer’s disease play a role in neurotoxicity to the hippocampus and promote oxidative stress, leading to depression, neurodegeneration, and cognitive decline [41]. Although these factors were not measured in this study, the obtained intervention effects may be explained by these mechanisms.

The effectiveness of the intervention is shown by the change in the level of sleep disorders. For the intervention group, the PSQI sleep disturbance score decreased by nearly half, from 10.2 to 5.6 points. For the control group, this trend is not clear; the PSQI disorder score decreased slightly from 9.0 points to 8.3 points. Comparing the results of the study with the pharmacological intervention of Naharci [42], the authors compared the total PSQI sleep quality scores of 78 dementia patients with and without sleep disorders prescribed Donepezil, Galantamine, or Rivastigmine, and a control group not taking anticholinesterase drugs. The results showed no significant changes in PSQI total scores between groups [42]. Another study conducted by Kouzuki in 2020 used aromatherapy to affect sleep quality in patients with cognitive disorders and dementia [43]. The study was a randomized controlled trial of aromatherapy as bath salts, to determine its effects on olfactory function, cognitive function, and sleep quality as determined by the PSQI-J (Japanese version of PSQI). Thirty-five participants with cognitive impairment and Alzheimer’s were randomized to one of three aromatherapy concentrations (0.1%, 0.5%, and 1%) with baths performed every night for 24 weeks. The results showed that, with aromatherapy, no significant changes in PSQI-J sleep quality were observed throughout the study period in all groups [43].

Compared to a randomized controlled trial conducted in 2020, Lina Wang implemented an exercise program in participants with cognitive impairment [44]. A total of 111 participants completed the study. Total PSQI scores were non-significantly lower in the intervention group at 12 weeks post-intervention, and then significantly lower at 24 weeks with a mean difference of −2.45 (95% CI: (−3.51)–(−1.39)), statistically significant with *p* = 0.001 [44]. Compare the results of the study with the work of Naismith in 2018 when conducting the program “Sleep well–Think well” [45]. This was a randomized controlled clinical trial of 35 people with cognitive impairment but no sleep disorders, providing five booklets. The evaluation of the results through the total PSQI score showed that the intervention group had an average change of −2.4 [45].

Our study showed that non-pharmacological multifactorial intervention was effective on autonomy in daily activities in dementia patients. Compared with the results of a study using the drug Rivastigmine combined with Citicoline, it showed the scores of the ADL basic functional life assessment scale and the daily living function assessment scale using the method tool. The IADL means had no significant difference between the intervention and control groups [46]. This partly demonstrates the benefits and explains the reason why non-pharmacological interventions are increasingly popular and of interest. A systematic review of research on the effects of combined physical and cognitive interventions found small to moderate positive effects on cognitive function in elderly patients with mild cognitive impairment and dementia [47]. Interventions combining both physical activity and cognitive training were cognitively beneficial in dementia patients (SMD = 0.36, 95% CI: 0.12–0.60, *p* < 0.05) and similar to mild cognitive impairment (SMD = 0.39, 95% CI: 0.15–0.63, *p* < 0.05). In particular, the analysis also showed a moderate to large positive effect after combining physical and cognitive interventions on activities of daily living (ADL) (SMD = 0.65, 95 CI %: 0.09–1.21, *p* < 0.01) [47]. Improving ADL scores will help increase autonomy in daily life, thereby also improving the quality of life of dementia patients [47]. 

Our study showed that quality of life scores tend to indicate the effectiveness of the intervention. For the intervention group, quality of life scores increased by 0.17 ± 0.19 points. In contrast, this score decreased in the control group with a score of 0.20 ± 0.30. To improve cognitive effectiveness, the current trend is to combine physical and cognitive interventions in dementia patients in groups—a miniature society [8]. A study published in 2021 to evaluate the effects of a group-based physical and cognitive intervention in elderly patients with dementia on quality of life in a healthcare service facility for geriatric health [48]. The eight-week intervention included group exercise and cognitive stimulation, twice per week, for 45 min each session. Twenty-five participants (ten in the control group and fifteen in the intervention group) were assessed pre–post. The analysis showed a significant difference in quality of life scores (F = 9.74, *p* = 0.006; maintained in the control group compared with an improving trend in the intervention group) [48]. While the control group showed a significant decrease in surrounding concern, the intervention group showed an increase in helping behavior. Track records show that the intervention group of patients talked to people around them more, said more positive words, increased contact with friends and family, appeared friendlier, and encouraged people in the group. The group continued to participate in the intervention [48]. Our research organizes classes at commune health stations—the basic health level, which is the foundation and backbone of the Vietnamese health system. Strengthening the grassroots health network and promoting primary healthcare is a priority in the health development policy in Vietnam [49].

Several limitations of this study should be taken into account. First, the study’s sample size is small due to the challenges of recruiting participants for interventions during the ongoing COVID-19 pandemic in Vietnam. Second, our study did not include the probable etiology of the dementia syndromes in the analysis, which could potentially introduce bias when compared with other studies that consider non-pharmacological interventions or anticholinesterase drugs. Vietnam’s healthcare system, within a country with a relatively low average income, exhibits limited awareness of the significance of dementia.

## 5. Conclusions

Our initial data have indicated that a non-pharmacological multifactorial intervention program, including physical activity, cognitive training, and participation in educational lectures in patients with dementia, has been shown to be potentially effective in contributing to cognitive improvement, including the memory field. Future research should explore the possibility of scaling up the intervention to reach a broader elderly population with dementia. Additionally, it is crucial to identify the likely causes of the dementia syndromes before implementing any intervention strategies.

## Figures and Tables

**Table 1:** General characteristics.

	Intervention Group (N = 44) Count (%)	Control Group (N = 44) Count (%)	*p*-Values
Gender			0.58
Male	9 (20.5)	7 (15.9)	
Female	35 (79.5)	37 (84.1)	
Age (years)—mean ± SD	68.5 ± 5.2	71.1 ± 7.96	0.07
Occupation before retirement			0.4
State agencies	8 (18.2)	5 (11.4)	
Farmer	32 (72.7)	31 (70.5)	
Other (workers, freelancers, etc.)	4 (9.09)	8 (18.2)	
Marital status			0.4
Married and living together	37 (84.1)	34 (77.3)	
Widowed/single/divorced	7 (15.9)	10 (22.7)	
Degree of dementia (score)—mean ± SD	1.1 ± 0.3	1.07 ± 0.3	0.44
Level of physical activity			0.6
Short	0 (0.0)	1 (2.3)	
Fit	28 (63.6)	28 (63.6)	
High	16 (36.4)	15 (34.1)	
30 s standing test (seconds)—mean ± SD	10.3 ± 4.0	12 ± 6.1	0.1
Time to get up and go (TUG) test (seconds)—mean ± SD	15.5 ± 5.3	14.2 ± 4.1	0.2
6 min walk test (m)—mean ± SD	109 ± 53.4	132 ± 72.2	0.09

**Table 2:** Changes in mental health in the intervention group and in the control group.

		Group		*p*-Values _(Intervention-control)_
		Intervention	Control	
		(Mean ± SD)		
Depression GDS-15 (score)	Before	4.8 ± 2.0	4.5 ± 2.6	<0.001
	After	2.9 ± 1.7	5.8 ± 2.5	
	*p*-value_Pre-post_	<0.001 *	0.02 *	
	Index changes	−1.9 ± 2.4	1.3 ± 3.7	
PSQI sleep disorders (score)	Before	10.2 ± 3.1	9.0 ± 3.7	<0.001 *
	After	5.6 ± 3.1	8.3 ± 2.8	
	*p*-value_Pre-post_	<0.001 *	0.3	
	Index changes	−4.6 ± 4.2	−0.7 ± 4.5	

*: statistically significant with *p* < 0.05; rank-sum test.

**Table 3:** Change in level of daily functioning.

		Group		*p*-Values _(Intervention-control)_
		Intervention	Control	
		(Mean ± SD)		
ADL	Before	6.41 ± 1.97	7.0 ± 1.3	<0.001 *
	After	7.64 ± 0.92	6.0 ± 1.9	
	*p*-value_Pre-post_	<0.001 *	0.007 *	
	Index changes	1.23 ± 1.75	−0.98 ± 2.19	
IADL	Before	4.86 ± 1.39	5.8 ± 0.7	<0.001 *
	After	5.89 ± 0.32	4.9 ± 1.2	
	*p*-value_Pre-post_	<0.001 *	<0.001 *	
	Index changes	1.02 ± 1.32	−0.93 ± 1.2	
EQ-5D-5L Quality of Life (score)	Before	0.64 (0.58; 0.85)	0.82 (0.65; 0.88)	<0.001 *
	After	0.92 (0.72; 0.97)	0.58 (0.55; 0.69)	
	*p*-value_Pre-post_	<0.001 *	<0.001 *	
	Index changes	0.17 ± 0.19	−0.20 ± 0.30	

*: statistically significant with *p* < 0.05; Ranksum test.

## Data Availability

All data generated or analyzed during this study are included in this article. Further inquiries can be directed to the corresponding author.

## References

[1] WHO (2019). Risk Reduction of Cognitive Decline and Dementia: WHO Guidelines.

[2] GBD 2019 Dementia Forecasting Collaborators (2022). Estimation of the global prevalence of dementia in 2019 and forecasted prevalence in 2050: An analysis for the Global Burden of Disease Study 2019. Lancet Public Health.

[3] Prince M., Comas-Herrera A., Knapp M., Guerchet M., Karagiannidou M. (2016). World Alzheimer Report 2016: Improving Healthcare for People Living with Dementia: Coverage, Quality and Costs Now and in the Future. Doctoral Dissertation.

[4] Van Minh H., Huong D.L., Giang K.B. (2008). Self-reported chronic diseases and associated sociodemographic status and lifestyle risk factors among rural Vietnamese adults. Scand. J. Public Health.

[5] Nam U.V., Duc N.M. (2021). Population Ageing and Older Persons in Viet Nam.

[6] Grandmaison E., Simard M. (2003). A critical review of memory stimulation programs in Alzheimer’s disease. J. Neuropsychiatry Clin. Neurosci..

[7] Ito Y., Urakami K. (2012). Evaluation of dementia-prevention classes for community-dwelling older adults with mild cognitive impairment. Psychogeriatrics.

[8] Kouzuki M., Kato T., Wada-Isoe K., Takeda S., Tamura A., Takanashi Y., Azumi S., Kojima Y., Maruyama C., Hayashi M. (2020). A program of exercise, brain training, and lecture to prevent cognitive decline. Ann. Clin. Transl. Neurol..

[9] Lamb S.E., Sheehan B., Atherton N., Nichols V., Collins H., Mistry D., Dosanjh S., Slowther A.M., Khan I., Petrou S. (2018). Dementia And Physical Activity (DAPA) trial of moderate to high intensity exercise training for people with dementia: Randomised controlled trial. BMJ.

[10] Woods B., Rai H.K., Elliott E., Aguirre E., Orrell M., Spector A. (2012). Cognitive stimulation to improve cognitive functioning in people with dementia. Cochrane Database Syst. Rev..

[11] Ngandu T., Lehtisalo J., Solomon A., Levälahti E., Ahtiluoto S., Antikainen R., Bäckman L., Hänninen T., Jula A., Laatikainen T. (2015). A 2 year multidomain intervention of diet, exercise, cognitive training, and vascular risk monitoring versus control to prevent cognitive decline in at-risk elderly people (FINGER): A randomised controlled trial. Lancet.

[12] Khanh D.V.D., Van Thang V., Dung H., BinhThang T. (2015). Prevalence of dementia among the elderly and health care needs for people living with dementiain an urban community of central Vietnam. Vietnam. J. Public Health.

[13] Nguyen V.T., Quach T.H.T., Pham A.G., Tran T.C. (2019). Feasibility, reliability, and validity of the Vietnamese version of the Clinical Dementia Rating. Dement. Geriatr. Cogn. Disord..

[14] Vietnam (2019). Hai Duong–Spotlight of Northern Key Economic Region. https://en.vietnamplus.vn/hai-duong-spotlight-of-northern-key-economic-region/160927.vnp.

[15] Tran D., Nguyen H., Pham T., Nguyen A.T., Nguyen H.T., Nguyen N.B., Nguyen B.H., Harvey D., Gitlin L., Hinton L. (2022). Resources for Enhancing Alzheimer’s Caregiver Health in Vietnam (REACH VN): Study protocol for a cluster randomized controlled trial to test the efficacy of a family dementia caregiver intervention in Vietnam. Trials.

[16] First M.B. (2013). Diagnostic and statistical manual of mental disorders, 5th edition, and clinical utility. J. Nerv. Ment. Dis..

[17] Vietnam Alzheimer Disease & Neurocognitive Disorders Association (2018). Dementia Diagnosis and Treatment Guidelines.

[18] Lovibond S.H., Lovibond P.F. (1996). Manual for the Depression Anxiety Stress Scales.

[19] Le T.A., Dang A.D., Tran A.H.T., Nguyen L.H., Nguyen T.H.T., Phan H.T., Latkin C.A., Tran B.X., Ho C.S.H., Ho R.C.M. (2019). Factors Associated with Sleep Disorders among Methadone-Maintained Drug Users in Vietnam. Int. J. Environ. Res. Public Health.

[20] Instrument: AUDIT-C Questionnaire. https://cde.drugabuse.gov/instrument/f229c68a-67ce-9a58-e040-bb89ad432be4.

[21] Tran B.X., Nguyen H.L.T., Le Q.N.H., Mai H.T., Ngo C., Hoang C.D., Nguyen H.H., Le H.Q., Van Nguyen H., Le H.T. (2018). Alcohol and tobacco use among methadone maintenance patients in Vietnamese rural mountainside areas. Addict. Behav. Rep..

[22] Tran D.V., Lee A.H., Au T.B., Nguyen C.T., Hoang D.V. (2013). Reliability and validity of the International Physical Activity Questionnaire-Short Form for older adults in Vietnam. Health Promot. J. Aust..

[23] Tran V.D., Do V.V., Pham N.M., Nguyen C.T., Xuong N.T., Jancey J., Lee A.H. (2020). Validity of the International Physical Activity Questionnaire-Short Form for Application in Asian Countries: A Study in Vietnam. Eval. Health Prof..

[24] Heatherton T.F., Kozlowski L.T., Frecker R.C., Fagerstrom K.O. (1991). The Fagerström test for nicotine dependence: A revision of the Fagerstrom Tolerance Questionnaire. Br. J. Addict..

[25] Pomerleau C.S., Carton S.M., Lutzke M.L., Flessland K.A., Pomerleau O.F. (1994). Reliability of the Fagerstrom tolerance questionnaire and the Fagerstrom test for nicotine dependence. Addict. Behav..

[26] Shelley D., Kumar P., Lee L., Nguyen L., Nguyen T.T., VanDevanter N., Cleland C.M., Nguyen N.T. (2017). Health care providers’ adherence to tobacco treatment for waterpipe, cigarette and dual users in Vietnam. Addict. Behav..

[27] Mai V.Q., Sun S., Minh H.V., Luo N., Giang K.B., Lindholm L., Sahlen K.G. (2020). An EQ-5D-5L Value Set for Vietnam. Qual. Life Res..

[28] Nourhashémi F., Andrieu S., Gillette-Guyonnet S., Vellas B., Albarède J.L., Grandjean H. (2001). Instrumental activities of daily living as a potential marker of frailty: A study of 7364 community-dwelling elderly women (the EPIDOS study). J. Gerontol. A Biol. Sci. Med. Sci..

[29] Nguyen T.T.H., Nguyen A.T., Vu T.T., Dau N.T., Nguyen P.Q., Nguyen T.X., Nguyen T.N., Nguyen H.T.T., Pham T., Vu H.T.T. (2021). Association of Frailty Status and Functional Disability among Community-Dwelling People Aged 80 and Older in Vietnam. BioMed Res. Int..

[30] Katz S., Ford A.B., Moskowitz R.W., Jackson B.A., Jaffe M.W. (1963). Studies of illness in the aged: The index of ADL: A standardized measure of biological and psychosocial function. JAMA.

[31] Hoi L.V., Thang P., Lindholm L. (2011). Elderly care in daily living in rural Vietnam: Need and its socioeconomic determinants. BMC Geriatr..

[32] Vinkers D.J., Gussekloo J., Stek M.L., Westendorp R.G., Van Der Mast R.C. (2004). The 15-item Geriatric Depression Scale (GDS-15) detects changes in depressive symptoms after a major negative life event. The Leiden 85-plus Study. Int. J. Geriatr. Psychiatry.

[33] Vu H.T.T., Lin V., Pham T., Pham T.L., Nguyen A.T., Nguyen H.T., Nguyen T.X., Nguyen T.N., Nguyen H.T.T., Nguyen T.T.H. (2019). Determining risk for depression among older people residing in Vietnamese rural settings. Int. J. Environ. Res. Public Health.

[34] Baharudin A.D., Din N.C., Subramaniam P., Razali R. (2019). The associations between behavioral-psychological symptoms of dementia (BPSD) and coping strategy, burden of care and personality style among low-income caregivers of patients with dementia. BMC Public Health.

[35] Peters M.E., Schwartz S., Han D., Rabins P.V., Steinberg M., Tschanz J.T., Lyketsos C.G. (2015). Neuropsychiatric symptoms as predictors of progression to severe Alzheimer’s dementia and death: The Cache County Dementia Progression Study. Am. J. Psychiatry.

[36] Fauth E.B., Gibbons A. (2014). Which behavioral and psychological symptoms of dementia are the most problematic? Variability by prevalence, intensity, distress ratings, and associations with caregiver depressive symptoms. Int. J. Geriatr. Psychiatry.

[37] Watt J.A., Goodarzi Z., Veroniki A.A., Nincic V., Khan P.A., Ghassemi M., Lai Y., Treister V., Thompson Y., Schneider R. (2021). Comparative efficacy of interventions for reducing symptoms of depression in people with dementia: Systematic review and network meta-analysis. BMJ.

[38] Paillard T., Rolland Y., de Souto Barreto P. (2015). Protective effects of physical exercise in Alzheimer’s disease and Parkinson’s disease: A narrative review. J. Clin. Neurol..

[39] Wang R., Holsinger R.D. (2018). Exercise-induced brain-derived neurotrophic factor expression: Therapeutic implications for Alzheimer’s dementia. Ageing Res. Rev..

[40] Corazza D.I., Sebastiao E., Pedroso R.V., Almeida Andreatto C.A., de Melo Coelho F.G., Gobbi S., Teodorov E., Santos-Galduroz R.F. (2014). Influence of chronic exercise on serum cortisol levels in older adults. Eur. Rev. Aging Phys. Act..

[41] Ouanes S., Popp J. (2019). High cortisol and the risk of dementia and Alzheimer’s disease: A review of the literature. Front. Aging Neurosci..

[42] Naharci M.I., Ozturk A., Yasar H., Cintosun U., Kocak N., Bozoglu E., Tasci I., Doruk H. (2015). Galantamine improves sleep quality in patients with dementia. Acta Neurol. Belg..

[43] Kouzuki M., Kitao S., Kaju T., Urakami K. (2020). Evaluation of the effect of aroma oil as a bath salt on cognitive function. Psychogeriatrics.

[44] Wang L., Wu B., Tao H., Chai N., Zhao X., Zhen X., Zhou X. (2020). Effects and mediating mechanisms of a structured limbs-exercise program on general cognitive function in older adults with mild cognitive impairment: A randomized controlled trial. Int. J. Nurs. Stud..

[45] Naismith S.L., Pye J., Terpening Z., Lewis S., Bartlett D. (2019). “Sleep Well, Think Well” Group Program for Mild Cognitive Impairment: A Randomized Controlled Pilot Study. Behav. Sleep. Med..

[46] Castagna A., Cotroneo A.M., Ruotolo G., Gareri P. (2016). The CITIRIVAD study: CITIcoline plus RIVAstigmine in elderly patients affected with dementia study. Clin. Drug Investig..

[47] Karssemeijer E.E., Aaronson J.J., Bossers W.W., Smits T.T., Kessels R.R. (2017). Positive effects of combined cognitive and physical exercise training on cognitive function in older adults with mild cognitive impairment or dementia: A meta-analysis. Ageing Res. Rev..

[48] Tanaka S., Yamagami T., Yamaguchi H. (2021). Effects of a group-based physical and cognitive intervention on social activity and quality of life for elderly people with dementia in a geriatric health service facility: A quasi-randomised controlled trial. Psychogeriatrics.

[49] Nguyen T.K.T. (2016). Joint Annual Health Review 2015: Strengthening Primary Health Care at the Grassroots towards Universal Health Coverage.

[50] ClinicalTrials.gov (2022). Effectiveness of Non-Pharmacological Interventions for Dementia among Elderly in Hai Duong Province, Vietnam. https://clinicaltrials.gov/ct2/show/NCT05351723.

